# Strategies for improving mental health and wellbeing used by adults ageing with HIV: a qualitative exploration

**DOI:** 10.12688/wellcomeopenres.18212.1

**Published:** 2022-08-26

**Authors:** Patrick N. Mwangala, Ryan G. Wagner, Charles R. Newton, Amina Abubakar

**Affiliations:** 1 Centre for Geographic Medicine Research Coast, Kenya Medical Research Institute (KEMRI), Kilifi, P O Box 230-80108, Kenya; 2 School of Public Health, University of the Witwatersrand, Johannesburg, 27 St Andrews Road, Parktown 2193, South Africa; 3 Institute for Human Development, Aga Khan University, Nairobi, P.O. BOX 30270-00100, Kenya; 4 MRC/Wits Rural Public Health and Health Transitions Research Unit (Agincourt), University of the Witwatersrand, Faculty of Health Sciences, Johannesburg, South Africa; 5 Department of Psychiatry, University of Oxford, Warneford Hospital, Warneford Ln, Oxford, OX3 7JX, UK; 6 Department of Public Health, Pwani University, Kilifi, P.O. BOX 195-80108, Kenya

**Keywords:** HIV; ageing; Kenya; coping; mental health and well-being

## Abstract

**Background**: Physical and mental health problems are common among older adults living with HIV (OALWH). Adaptive coping strategies play a vital role in improving these adults' mental health and well-being despite the deleterious effects of HIV and ageing. However, in sub-Saharan Africa, limited evidence exists on the commonly utilized coping strategies in this population. We explore the coping strategies used by Kenyan OALWH to improve their mental health and wellbeing.

**Methods**: Semi-structured in-depth interviews were conducted between October and December 2019 with 56 participants: 34 OALWH (53% female), 11 healthcare providers (63% female) and 11 primary caregivers (73% female) in Kilifi County. All interviews were audio-recorded and transcribed verbatim. We used the framework approach to synthesize the qualitative data.

**Results**: Five major themes emerged from the analysis of participants’ narratives, including self-care practices, religion and spirituality, relational living (social connectedness), generativity, identity, and mastery. Our study further revealed maladaptive coping strategies, including reliance on over-the-counter medications, self-isolation, waiting to see if symptoms would subside despite doing nothing, and HIV treatment interruptions during prolonged periods of prayer and fasting.

**Conclusions**: Our findings provide an initial understanding of the coping strategies used by OALWH to confront HIV and ageing challenges in a low-literacy, low socio-economic Kenyan setting. Our results suggest that interventions designed to enhance personal capacity, social support, positive religiosity and spirituality, and intergenerational connections may be beneficial in improving the mental health and well-being of OALWH.

## Introduction

Population ageing is now a reality worldwide, and sub-Saharan Africa (SSA) has the most rapidly ageing population ^1^. Thus, ageing and health are global priorities considering the increasing and changing burden of disease due to the ageing population, the corresponding need for long-term healthcare systems to adapt to evolving demands, and the call to invest in healthy ageing ^2, 3^. One subpopulation of older adults that deserves special attention is that of adults ageing with HIV, aged ≥50 years. With improved access to combination antiretroviral therapy (cART) and a rising trend of HIV infection among the elderly, the number of older adults living with HIV (OALWH) in the world is expected to rise rapidly ^4^. This puts more pressure on HIV health services to develop and implement appropriate programs that address these adults' unique needs. Despite limited research on ageing with HIV, especially in SSA ^5, 6^, existing evidence suggests that apart from physical health challenges, OALWH face unique mental health burdens and challenges to their social well-being that may differ from their uninfected peers and younger counterparts, e.g. elevated comorbidity and early-onset geriatric conditions ^6– 11^. Despite the observed challenges, intervention research designed to address the unique needs of OALWH is insufficient ^12^, and it is unclear how these adults cope and age with HIV, especially in SSA, where the health system is largely unprepared to meet their complex healthcare needs. 

Based on one perspective in the existing literature on HIV and ageing ^6, 10^, it would appear that the outlook for OALWH is bleak despite the gains conferred by cART. Nonetheless, there is another approach to understanding the life experiences of OALWH – an approach focusing on strength and not on deficit ^13– 15^. The deficit or challenges approach, which focuses on monitoring the presence of chronic comorbidities in OALWH, has important limitations. It is disease-centred and measures a person’s health deficits at a stage in life where little can be done to prevent the decline in function. Moreover, the approach overlooks the fact that the process of ageing with HIV is multidimensional where, in addition to the impact of the virus on biological systems, other factors shape the ageing process, often well before an individual gets to middle age. The strengths approach encourages policymakers and health professionals to look beyond disease states and approach ageing with HIV as a process to maximize a person’s well-being by strengthening positive factors, e.g. adaptive coping skills.

As an intermediate mechanism of psychological stress and health outcomes, coping strategies are vital in maintaining physical and mental health ^16, 17^. The research on coping originates and is largely influenced by the work of Lazarus and Folkman ^18^. They define coping as the cognitive and behavioural efforts undertaken by an individual to alter or manage a problem caused by a specific stressful situation. According to Lazarus and colleagues, stress emanates from an imbalance between demands and resources or when pressure surpasses one’s perceived ability to cope ^18^.

While several studies have examined how people living with HIV (PLWH) cope with a chronic illness, few have addressed this issue among OALWH, especially in SSA ^17, 19^. Drawing on semi-structured interviews with 76 OALWH in the United Kingdom, Rosenfeld and colleagues identified a few coping strategies, including volunteering, accentuating the positive, minimizing the role of HIV medications, and accessing support from mental health workers and HIV organizations ^20^. In America, the frequently reported coping strategies for healthy ageing among OALWH include self-care, spirituality, social support (from family, friends, professionals, and pets), and generativity (e.g. mentoring, volunteerism) ^21– 25^. In SSA, little research has focused on how older adults cope with HIV and illness-related stressors. In one of the few studies on coping in this population in the region, OALWH in South Africa adopted HIV status acceptance, ART adherence, abstinence from sexual relations, and accessing social support as key coping strategies in dealing with HIV diagnosis, stigma, disclosure and the healthcare system ^26^.

In 2019, we conducted a qualitative study on the coast of Kenya to understand the health challenges and coping strategies of older adults living with HIV among 56 participants (34 OALWH, 11 caregivers and 11 healthcare providers). Arising from this dataset are two manuscripts; the first one (a preprint published in medRxiv) reports the health challenges facing OALWH ^27^. Briefly, the participants’ narratives indicated that mental and psychosocial complaints, e.g. common mental problems, poverty, stigma and discrimination) were common among the OALWH in addition to physical health challenges, e.g. comorbidities and somatic symptoms). The current manuscript is the second paper, where we explore the same participants' perceptions to understand the coping strategies and support available to the OALWH in this low-resource setting.

## Methods

### Ethics

Written informed consent was obtained from all respondents before participation. Ethical clearance was granted by the local institutional review board, the Kenya Medical Research Institute Scientific and Ethics Review Unit (KEMRI/SERU/CGMR-C/152/3804), on March 6 2019. Permission to conduct the study in Kilifi County was granted by the research office, Department of Health (HP/KCHS/VOL.X/171) on April 3 2019.

### Study setting and sample

This study was conducted towards the end of 2019 at the Kenya Medical Research Institute-Wellcome Trust Research Programme (KWTRP) in Kilifi County on the Kenyan coast. Kilifi is mainly rural, and the majority of the inhabitants belong to the Mijikenda tribe, whose primary source of livelihood is small scale trading and subsistence farming. More than half of Kilifi residents live below the poverty line, and a significant proportion (about 40%) do not have formal education ^28^. The HIV prevalence is higher among females (54 per 1000 people) than males (23 per 1000 people) ^29^.

Our sample included: a) older adults living with HIV aged ≥50 years receiving HIV care and treatment at the Kilifi County Referral Hospital (KCH) HIV clinic; b) primary caregivers of older adults living with HIV and c) healthcare providers attending to the older adults living with HIV including nurses, clinical officers, counsellors, and project coordinators of community-based organizations.

### Sampling strategy and recruitment

We recruited our sample using a purposive sampling technique to maximize diversity in respondents' characteristics, e.g. age and sex. Participants were recruited by trained study staff in liaison with a community health volunteer stationed at the KCH specialized HIV clinic. After introducing the study at the clinic, OALWH were approached (during their routine clinic visits), and those expressing interest were given more information regarding the project and how to participate. We had no specific target sample size. We recruited and assessed participants until we reached data saturation. The older adults living with HIV had to be at least 50 years old and on cART for them to take part. Caregivers were identified through the OALWH during their routine clinic visits. To participate, they had to be directly involved in the care of an OALWH. We approached healthcare providers at their places of work and invited them to participate. We targeted providers who were directly involved in the care of OALWH. Participants who agreed to take part were interviewed once, immediately after enrolment, if available, or at an appointment made for a future date. Apart from a few health providers who were interviewed at their place of work, the majority (over 80%) were interviewed at the KEMRI Wellcome Trust Research Programme. Only the interviewing researcher and participant were present during the interview. The researcher and participants had no prior contact/relationship in this study.

### Data collection and instruments

The lead author (PNM) conducted all the qualitative interviews, which lasted approximately 45 – 60 minutes each. At the time of conducting this study, PNM was a PhD Fellow at the KEMRI-Wellcome Trust Research Programme, Kilifi, Kenya, with extensive training in mixed methods. A pre-tested, semi-structured interview was used to guide the interviews. The interview guide was created new for this study. Over three-quarters of the interviews were conducted in Swahili – the national language in Kenya. The rest of the interviews were conducted in either Giryama (the local language) or English. All interviews were digitally recorded with permission from the participants.

General information about coping/living strategies and social support was elicited among participants, leading to specific explorations of coping with long-term HIV, ageing, and health changes, e.g. mental difficulties. Interview questions focused on what challenges participants were experiencing (for OALWH) or presented with (for the other key informants) regarding ageing with HIV, what helped the OALWH through difficult periods, and how they coped daily and long-term basis. The interviewing researcher took down some notes during and after the interview, which helped plan subsequent interviews. 

### Data analysis

The audio-recorded interviews were transcribed verbatim by four research assistants. QSR Nvivo software version 11.0 was used for data management and analysis. The analysis of our qualitative data followed the Framework approach as described by Ritchie and Spice ^30^. A preliminary coding framework was developed inductively (through in-depth reading of transcripts) and deductively (considering themes in the interview guide) by two authors (PNM and AA). This was done independently by the two authors. The arising codes were then discussed, and a consensus was reached on how they should be brought together into themes. The initial coding framework was subsequently expanded to capture any emerging theme as coding progressed. After coding, themes related to specific concepts were grouped to form categories and later exported to a word document to produce charts. The charts were then used to summarize the data, looking for similarities or differences. 

## Results

### Summary of participant characteristics

We included 56 participants in this study (34 OALWH, 11 healthcare providers and 11 primary caregivers) after reaching data saturation. All approached participants agreed to participate. Among OALWH, slightly more than half were women (53%); most (82%) had up to a primary level of education, most (73%) lived in multigenerational households, and had a median age of 57 years (54 – 63 years). All the OALWH were on HIV treatment and had lived with HIV for a median duration of 12 years (10 – 15 years). Healthcare providers comprised six registered nurses, two clinical officers, two project managers of community-based organizations and one HIV counsellor. On the other hand, all the primary caregivers were family members, the majority (73%) of whom were female. Further sociodemographic information and HIV-related characteristics of the OALWH are described elsewhere ^27^.

### Perceived coping strategies for mental and well-being challenges

Participants utilized multiple strategies to maintain or improve their mental health and well-being in the face of various biopsychosocial challenges. Analysis of the respondents’ narrative accounts revealed five major themes: self-care, religion and spirituality, generativity, social connectedness, identity, and mastery.

### Self-care

Within the respondents’ narratives, this was either a change in behaviour or a pattern that developed over time to cope and live with HIV. The frequently discussed self-care strategies included abandoning/abstaining from risky behaviours (e.g. multiple sex partners, unprotected sex, tobacco smoking, and hazardous alcohol use), strict treatment adherence (e.g. cART and routine HIV clinic appointments), acceptance and disclosure of one’s HIV status, shifting focus (from negative to positive things), and practising self-control (e.g. managing thoughts or attitudes). To a lesser extent, some OALWH reported monitoring their diet (as a form of weight loss strategy), seeking information (e.g. from seminars and HIV clinics), and using traditional forms of treatment to stay healthy. Similarly, some OALWH engaged in physical exercise, including walking, jogging, running, swimming, skipping rope, traditional dances, and makeshift weightlifting. However, many of these activities were unstructured and irregular, while some of the OALWH (especially women) felt embarrassed doing some of the activities, e.g. swimming and weightlifting. Still, a few of the OALWH acknowledged that their nature of work (e.g. walking long distances to work; heavy manual work - such as pulling carts, casual labourer at construction sites; farming, grazing livestock, and house chores) was as good as physical exercise. Common sources of information for these strategies were trial and error and sometimes healthcare providers, family, and friends. A good number of the self-care strategies employed were regarded as helpful, e.g. reduced distress and better physical health outcomes. 

Other self-care strategies included relying on over-the-counter medications (especially for symptoms like pain and sleeping difficulties) and skipping HIV medications when one lacks food. *Waiting* to see if the symptoms would lessen despite doing nothing was also practised by a few OALWH. Some of the participants’ quotes on this theme are provided in Table 1.

**Table 1.** Participants' quotes on perceived coping strategies for mental and well-being challenges.

Main coping strategy	Participant’s quote
***Selfcare***	“Letting go of certain behaviours has helped me a lot. I wasted much money on drinking sprees, multiple sexual partners who, of course, needed upkeep, and other pleasures. However, I abandoned all these things following my diagnosis, and I can see some improvements in my health and life”. **(** ***Male OALWH*** )
	“I maintain a very active life! I usually wake up early in the morning, make my bed, mop my house, prepare breakfast, take a shower, and leave for work. I have a niece staying with me, but I do most of the work myself. I wash my clothes after every two days. I also do farming. I tend my greenhouse and chicken. I wash the dishes in the morning. I am very happy”. **(** ***Female OALWH*** )
	“I usually wake up very tired and having muscular pains! I have to take *action* ( *a common analgesic*) to work and be productive. It is frustrating because I have to buy medications every day. My well-being is dependent on these medications”. **(** ***Female OALWH*** )
	“Some of the clients stop taking their ART medications. Some usually say, “I found a herbal doctor who said my disease will go away if I use their herbal medication.” So they will stop the ART medication until they realize that the herbal medicine is not helping and then come back to the clinic, usually in worse off conditions”. ( ***Clinical officer***)
***Religion and*** *** spirituality***	“When I find myself crowded with thoughts, as I shared with you initially, I usually run to God because he is the one who knows why I am undergoing those issues. And when I pray, I usually feel comforted, relieved and at peace even if I still do not have anything …but I feel much better when I put God first”. **(** ***Female OALWH***)
	“Sometimes I am worried, stressed, anxious and experience nightmares, but I encourage myself in God. I usually kneel and pray to God. I wake up my wife, we pray together and go back to sleep, and we usually feel much better in the morning.” **(** ***Male OALWH*** )
	“Sometimes back, one of my kidneys had problems…it was quite painful. Later, as I was studying the bible, I was instructed ( *by God)* to go for prayers for 18 days. I left home and camped in a nearby bush for 18 days, and that was the end of that problem. I have never had that issue again; I never went to a hospital. You know God does not need help to do his work…he is perfect!” **(** ***Male OALWH*** )
	“In our community, most of them go to traditional doctors or *waombezi* (seers or prophets) in addition to their routine clinics. And some of them stop taking their ART medications. For instance, one may be instructed to leave their ART medications and pray for three months or sometimes drink some traditional drugs for a certain duration. So it becomes complicated; they usually come back in worse conditions.” ( ***Clinical officer***)
***Generativity***	“I usually volunteer at the clinic as a peer counsellor. I have had a few clients come to me looking for marriage partners. I usually go ahead and connect them. As a matter of fact, I have succeeded in joining five people who are now happily married”. **(** ***Female OALWH*** )
	“On several occasions, the clinicians have called upon me to talk to my peers on different issues, e.g. stigma and non-suppressed HIV”. **(** ***Female OALWH*** )
	“My role as a counsellor or an elder happens on different platforms…at the clinic; I may advise people on various issues, including adherence to ART. I also like discussing and sharing things with community members, my children…e.g. on different issues such as developments, health, and well-being. I love it very much”. **(** ***Male OALWH*** )
	“I have one person who is their…like their head…So in case, I find a client with stigma …I call him, And he talks with the client…I find him very helpful”. ( ***Nursing officer*** )
	“She ( *OALWH*) helps me a lot. For instance, she educates women in church on various issues relating to HIV…she also teaches children and girls… let us say she has the qualities of a leader”. ( ***Caregiver*** )
**Social ** **connectedness/** **support systems**	“It affects me a lot ( *living with HIV*)…for instance, we slept without taking supper yesterday. I had to borrow the fare to come here today. However, it is not every day that we sleep hungry because I have a good friend who owns a hotel nearby…he usually gives us food. Sometimes I just take the children to eat there and pay later. He encourages me not to be worried”. ( ***Male OALWH**)*
	I usually encourage, advise and motivate him so that he can bounce back to his normal well-being. I always try to understand him…be empathetic so that he can recover completely. I also help him with food, and even other people support him with food…however, most of the time, we counsel him”. ( ***Caregiver***)
	“I did not see the importance (of going to support groups)…why spend hours listening to people…it was even depressing…every time people are saying the same things. People were complaining a lot…there were no benefits…you know sometimes you want to see tangible benefits, e.g. we get registered, look for support such as financial support, but there was nothing like that. I like a positive outlook”. ( **Female OALWH**)
	“Financial problem is a big issue among these adults…there is one I had to give some little income [ *about $10]* to start selling fish…because I had to ask her what she could do…then she told me that she used to sell fish. So I gave her 1000 Ksh [ *about $10]* to start her small business. Since that day, I have never had a problem with her routine clinic appointments. I thank God. Even her overall health has improved”. ( ***Nursing officer**)*
	“We have five areas of empowerment, including spiritual, economic, emotional, social and physical aspects. We support them in all these areas…our primary goal is to help them stand on their own”. ( ***Project Manager CBO***)
**Identity and** ** mastery**	“To be honest, my brother…I do not despair easily…I have trained myself that way…I know I was born one day, and there is an appointed time for me to die! I have made up my mind to do everything I can to live today, and when my time to die comes, I will say goodbye! As long as I am alive, I will take my medications. I am the last person to surrender! I am like a soldier”. ( ***Male OALWH** )*
	“I did not plan on getting this disease…so I have chosen to take each day at a time…I have not aged because of HIV…as a matter of fact, I am least concerned about the HIV infection…I have forgotten about it! I believe that as long as I take my medications well, I will continue living like anyone else…if I follow the clinician’s advice. That is my philosophy, and I am not worried at all!” ( ***Female OALWH** )*
	“The cohort I have is very informed about their medications and health. They even carry their medications everywhere. They tell you that ‘this medication is my life’”. ( ***Nursing officer***)
	“I have not received help or support from people, and I do not bother to ask! I like taking care of myself…I do not want to make this disease a source of income by depending on others…I do my own business…not big, but I can live off my sweat… I have learnt to go out of my way and be active, and hardworking. I have realized this is important for my health and well-being as well. I also eat well and do some exercises…mostly walking”. (Female OALWH)

### Religion and spirituality

Most respondents described religion or spiritual practices as an important strategy through which OALWH drew their strength, besides giving them hope to live. Prayers, belief in God, attending religious meetings, singing, or listening to worship music, reading the scriptures or listening to uplifting messages of hope, fellowship with other believers, membership to church groups and other expressions of spirituality were regarded as instrumental in providing OALWH with emotional, social and sometimes financial support. For instance, several OALWH reported using prayer when faced with persistent emotional challenges, e.g. thinking too much, sleeping problems, and having nightmares. Others sought emotional support (counselling) and material support (with basic needs) from pastors.

However, a few healthcare providers felt that some of the religious activities or beliefs among OALWH had negative implications on health, e.g. poor retention in care and virologic non-suppression. For instance, some of the OALWH (especially women) sought the services of religious people popularly known as “ *waombezi”,* believed to be seers or prophets with special healing powers. Subsequently, they stopped taking their ART drugs, believing they have been cured of HIV infection after receiving prayers from these seers, only for them to come back later to the HIV clinic with severe HIV disease. Others occasionally go into prolonged periods of prayer and fasting where they change their ART medication schedule or stop taking them altogether. Still, others mixed biomedical and traditional forms of treatment, which sometimes led to poor health outcomes in the OALWH.

### Generativity

Generativity is the willingness to engage in acts that promote the well-being of other people besides self and family, especially younger generations, to ensure their survival. This concept has its origin in the psychoanalyst Erik Erikson emphasizing a concern for establishing, caring, nurturing, guiding, and maintaining the next generations ^31^. Our analysis of participants’ narratives revealed three main categories of generativity. In the first category, many OALWH took leadership roles in the family and community, e.g. as family heads, village elders, religious leaders, and HIV support group coordinators. Through sharing stories of the years of the HIV epidemic, serving on different committees, and being involved with HIV advocacy, these respondents utilized their status, time and experience to lead others and, in the process, derived personal satisfaction and fulfilment. The second form of generativity discussed was volunteerism, where some of the OALWH offered their skills and services to benefit other individuals. Participants volunteered in different ways, including serving as community health volunteers. They offered various services such as health education to families of people living with HIV and caregiving to some people living with HIV. For many of these respondents, volunteering provided a focus, purpose, and opportunities to make a social contribution and reciprocate the support they had experienced. Given their extended duration of living with HIV, some participants acknowledged the need to serve as mentors to nurture and guide those recently diagnosed with HIV and young people living with HIV. This came out as the third form of generativity. 

### Social connectedness/support systems

Respondents’ narratives also uncovered various formal and informal support systems regarded as positive contributors to mental health and well-being. Support was received from diverse sources, including family members, friends, healthcare providers within the HIV clinics, HIV community organizations, and informal support groups. Family was an important source of comfort and support in managing the challenges of ageing with HIV for some of the OALWH. Specific support included occasional financial support (for fare and basic needs), companionship, medication and routine clinic appointment reminders, and emotional support. However, some of the OALWH felt more comfortable seeking help from other sources (e.g. friends and healthcare providers) other than the family partly because of dysfunctional families, fear of disclosing their HIV status, or simply because they lacked the means.

Several participants also portrayed existing mental health services (within HIV clinics) as especially important settings for formal and informal therapeutic conversations with people who would listen and support them, as well as providing specialist knowledge vital in the management of HIV and ageing challenges. Some of the mental health support services offered by HIV clinics included occasional psychoeducation on HIV, occasional screening of depressive symptoms, psychopharmacology (e.g. provision of antidepressants), peer counselling, referral services (with community organizations or other higher-level hospitals) and HIV support groups (e.g. for discordant couples). However, such services tended to vary from one HIV clinic to another. Specialist services, e.g., psychologists, psychiatrists, and geriatricians, were unavailable.

Community-based organizations dedicated to the health and well-being of OALWH did not come up in our discussions with the participants. The few existing organizations supported different groups of people living with HIV, including OALWH. Some of the support services they offered included occasional assistance with basic needs (e.g. food and tuition fees for orphans), supporting community health volunteers (e.g. fare), employing healthcare providers (e.g. counsellors), community HIV testing, empowering people living with HIV (spiritually, economically – e.g. through skills training including making soaps, tailoring, weaving, psychosocial counselling, referral services), and legal services (mainly for women).

Although the HIV support groups were perceived as instrumental in giving a sense of belonging, membership and camaraderie, which buffered or moderated many of the stresses of ageing with HIV, some of the OALWH avoided these groups or scaled back their involvement. To some of these adults, attending the groups undermined their goal of lessening HIV’s role in their current and imagined future, which they regarded vital for their mental health and well-being. Thus, they avoided the groups to limit a persistent focus on that issue. Other OALWH cited monotony, time-wasting, gossip, lack of support, poor leadership and no apparent benefits with such groups hence their collapse.

### Identity and mastery

Identity refers to how individuals perceive themselves in relation to HIV, while mastery is the ability to accomplish what needs to be done to live a good life. Identity can also be understood as HIV centrality, how much of oneself is defined by the HIV diagnosis. Many of the OALWH described themselves as much more than their diagnosis. The concepts of self-reliance, self-supporting, depending on oneself as a resource and managing one’s care incorporated behaviours and self-perceptions of mastery and control in ageing with HIV. According to several OALWH, identity and mastery developed over time. In the process, respondents utilized their background and personal strengths to identify tools to achieve mastery over the challenges of ageing with HIV. Several participants noted that they no longer had problems with their HIV status as they used to be in the early years of HIV diagnosis. As a matter of fact, they could disclose to anyone, including public gatherings. Others later came to the point of viewing their HIV status as a blessing in disguise or a wake-up call in their lives, making them gain control of their lives, e.g. through investments, personal development, abandoning risky behaviours such as multiple sexual partners and substance use. Some considered themselves better off than their uninfected peers who still engage in such behaviours. Attaining mastery also gave OALWH opportunities to engage in mentorship and psychoeducation, especially for newly diagnosed HIV individuals. 

## Discussion

### Summary of key findings

Given the demographic imperative of a rapidly ageing society and the growing number of older adults ageing with multiple chronic conditions such as HIV, there has been rising attention in recent years on understanding ways to promote resilience among older adults and on designing interventions that can strengthen their ability to cope, improve quality of life, prevent, or delay functional decline, and decrease healthcare costs. This study provides a preliminary understanding of coping among OALWH residing in a low-resource setting in Kenya. Overall, our findings portray a picture of OALWH who integrate various living strategies into their lifestyle to address their mental health and well-being challenges. Five main strategies emerged: self-care practices, religion and spirituality activities, relational living (social connectedness), generativity, identity, and mastery. Many of these strategies were developed by the OALWH themselves, sometimes collaborating with other peers, providers, and caregivers, thus representing respondents’ deep engagement with their mental health and well-being and a culture of survival. This highlights the importance of working with OALWH to understand their values and aspirations and reinforces calls to include their subjective experiences in care and management. Our study also revealed maladaptive coping strategies among these adults highlighting the crucial need to identify such patterns and look for ways to address them, given their impact on health.

### Self-care

Although a well-established approach for several chronic conditions ^32, 33^, self-care programmes for OALWH are still emerging, especially in SSA. Self-care is regarded as a critical element in chronic care; thus, a major focus of many interventions and patients who engage in self-care have been shown to have significantly improved clinical outcomes, a better quality of life, fewer hospitalizations, and longer survival ^32, 34^. Participants discussed several self-care strategies to promote their mental health and well-being in the current study. Reported self-care practices ranged from those with beneficial health impacts to those potentially deleterious to their overall health. Although existing evidence provides only modest evidence of the effectiveness of these strategies, most of these strategies were reported to be effective in promoting mental health and well-being among OALWH. Many of the OALWH relied upon their internal strengths for continued adherence to HIV treatment and avoidance/abandoning of previously harmful lifestyle elements, including drug use. With time, several OALWH reported reduced family conflicts, improved self-efficacy, and better financial decisions (investments and planning for one’s old age). Although male and female OALWH acknowledged the importance of others, many observed that they were responsible for their self-preservation. Our findings of OALWH embracing their responsibility for self-preservation are consistent with those reported by Emlet and colleagues in the United States of America among 25 OALWH ^21^ and Solomon and colleagues in Canada among 14 older men living with HIV ^24^. 

### Religion and spirituality

Consistent with previous work, this study found that religion and spirituality played an essential role in coping among OALWH ^25^. In literature, involvement in religious and spiritual practices has been noted to correspond to better health-related outcomes ^25^. Such association may provide one with social support, norms for healthy behaviours, and a sense of hope and well-being, ultimately promoting their mental health ^35^. These strategies may also act as buffers to life stress by allowing them to interpret their life experiences in the context of their beliefs, which provide purpose and meaning in life and promote transcendence over circumstances. Nevertheless, certain religious practices may hinder positive biopsychosocial outcomes. In the current study, some of the healthcare providers noted that some of the OALWH abandoned their ART medications after receiving prayers from their pastors or after taking part in an extended duration of prayer and fasting. There is a need to re-examine the role that “traditional healers” and other “influencers” play in the identification and care of OALWH and how to work together to ensure that OALWH maintain their treatment routines.

### Social connectedness

While several studies of PLWH have identified the value of receiving social support ^36, 37^, few have examined this subject among OALWH. Understanding the influence of perceived social support on the mental health and well-being of OALWH is crucial because it is likely to play a major role in managing their multiple negative life stressors. Greater social connectedness may address the negative mental and physical impacts of ageing with HIV through enhanced cognitive efficiency, better social competency, productivity, personal control, and life satisfaction. Our results attest that OALWH are aware of the significance of others in their lives. Family members, sexual partners, friends, religious groups, and the HIV clinics were regarded as important sources of support by many OALWH. Similarly, Rosenfeld and colleagues attest to the importance of social support in improving the mental health and well-being of OALWH residing in the United Kingdom ^20^. Our findings also echo previous observations by Emlet and colleagues in the US ^21^. Interestingly, avoidance of HIV support groups was noted as a strategy to support mental health and well-being by a few OALWH by shifting the constant focus on HIV. This finding is consistent with previous findings in other places ^20, 24^. This underscores the need for HIV support groups to understand clients’ needs and fine-tune their support services accordingly. Although having others to rely upon for help and support, many OALWH maintained an aspect of independence to avoid overreliance on others who, according to them, had responsibilities and problems of their own. 

### Generativity

Another important element of coping for OALWH revealed in this study was generativity – a sense of concern for the well-being of others, especially future generations. Taking up leadership roles, volunteerism and mentorship were identified as important elements of generativity among OALWH. They were often associated with personal satisfaction and happiness and reduced the focus on their health challenges. Our findings reinforce the importance of social connectedness among OALWH but, more importantly, add a new element to previous research. Instead of viewing OALWH as the passive recipients of social support and care, it places the individuals in a contributory role of giving back to the community through these acts of generativity. Our findings align with those of Emlet and colleagues among 30 OALWH in Canada ^22^. Programmes that enhance intergenerational connections, community involvement and generative acts within the HIV community could potentially promote the mental health and well-being of OALWH. 

### Identity and mastery

Mastery has previously been examined in the HIV literature, and results show that acquiring mastery is essential in improving mental health and reducing symptoms of common mental disorders ^38, 39^. Our participants discussed how they achieved identity and mastery in their lives, and this was important in ageing with HIV. Many of them described their HIV diagnosis as a turning point in their lives and expressed relief (from the persistent unknown suffering before HIV diagnosis). After the initial shock was over, many of them drew on their backgrounds and strengths to identify tools to achieve mastery over the challenges of ageing with HIV. This ushered in a period of lifestyle adjustments, e.g. diet, risky behaviours, taking personal control of one’s life, and thinking of one’s future (e.g. investments). For some of these clients, the awareness of the complexities of ageing with HIV increases their urgency for mastery of self-care. Our findings mirror those of earlier research conducted among OALWH in high-income countries (HICs) ^40^. 

Apart from the positive highlights on living strategies, our study also reveals important concerns in the coping of OALWH in this setting which may threaten their mental health and well-being. Though not many, some of the OALWH reported over-reliance on over-the-counter medications. Overreliance on OTC drugs could be due to medications being the only option offered to these clients by their healthcare providers, health illiteracy on the part of caregivers, or financial difficulties in seeking other therapies. Still, some of the OALWH isolated themselves from sources of support, afraid of what people will say about them. Others tended to wait for symptoms to run their course or do ‘nothing’. Many of such symptoms were part of the symptom profile for common mental disorders suggesting undiagnosed or inadequately treated common mental disorders. The OALWH may not know how to relieve such symptoms or maybe lack the motivation to find a solution. This emphasizes the need for healthcare providers to adequately screen for such symptoms and have knowledge of the overlapping presentation of these disorders experienced by OALWH. The providers noted that they rarely screened for common mental disorders in these clients mainly because of competing interests (e.g. the treatment emphasis is predominantly on physical health outcomes such as HIV viral load suppression) and inadequate treatment modalities for common mental disorders (CMDs). Others mixed biomedical care with traditional therapies, many of which were deemed ineffective, leading to health deterioration among those affected. Still, other OALHW skipped their medications whenever they missed food. These observations highlight some of the well-known patient-related challenges (e.g. health illiteracy, culture, financial difficulties) as well as health system challenges (e.g. inadequately trained providers to screen for CMDs and inadequate resources such as essential medicines) ^41, 42^ which have been pointed out as some of the barriers in the delivery of chronic care and promotion of coping strategies in this population.

### Implications

Healthcare providers involved in the care of OALWH need to take a more holistic view of the healthcare of these adults and recognize that a major component of care is delivered by ‘self’. Patient-centred care and self-care are essential elements, particularly in chronic care. These strategies help decongest primary healthcare facilities and reduce frequent health visits for patients while maintaining continuity of care at the household and community levels. Our paper has highlighted the importance of self-care, the different forms it takes and potential opportunities to embrace some of these modalities further. From our present findings and the available evidence on some of these strategies, ignoring them could lead to poorer health outcomes and poorer care in this population ^17^. To attain more favourable mental health and well-being, OALWH require training and support to effectively cope with HIV and ageing challenges, communicate their potential needs to access services and participate in shared decision making with their providers. Given that the challenges of OALWH can be diverse-including medical, physical and social - and that individual preferences will often dictate uptake of and access to different models (e.g. new technologies, group work, individual therapy), theoretically, sound models of intervention should be considered to meet the preferences of various population groups. Additional qualitative studies could further examine how characteristics of resilience and strengths are utilized in HIV and ageing outcomes. Quantitative studies could shed some light on which subgroups of OALWH are more likely or less likely to cope effectively. These findings could lead to identifying at-risk individuals and developing targeted interventions. 

### Strengths and limitations

Our study is among the few reports in SSA and the first in Kenya to comprehensively explore the coping strategies for OALWH with mental health and well-being challenges. Unlike previous reports, an important strength of this work is that respondents included a diverse group of stakeholders, including clinicians, community-based organizations, primary caregivers, and the OALWH themselves. Nonetheless, our participants were predominantly from a rural setting and in routine HIV care; thus, their circumstances may differ from those in urban areas and those who may not be in routine HIV care. We minimized potential individual influences in the current study by maintaining reflexivity and frequent discussions with the research team to provide rigour and credibility.

## Conclusions

Our findings provide an initial understanding of the coping strategies utilized by OALWH to confront HIV and ageing challenges in a low-literacy Kenyan setting. This study underscores the importance of coping in this vulnerable population and suggests that interventions designed to enhance personal capacity, social support, positive religiosity and spirituality, intergenerational connections, self-identify, and mastery may be beneficial in the improvement of mental health and well-being in this growing population. Our study also highlights a few potential obstacles that may impede successful coping in this population, including over-reliance on over-the-counter medication, waiting for symptoms to run their course or doing ‘nothing’, self-isolation, seeking support in the wrong places, and skipping meals. Further engagement with traditional healers/prophets may be warranted to mitigate the present obstacles and dangers some of these individuals present to the OALWH. These findings will assist providers, caregivers, and other stakeholders to best address the specific health and well-being needs of OALWH in this setting.

## Data availability

### Underlying data

The underlying data transcripts are not publicly available due to confidentiality reasons. However, individual applications can be made through the data governance committee of the KEMRI Wellcome Trust Research Programme, which will review the application and advise as appropriate. Requests can be sent to the coordinator of the Data Governance Committee using the following email dgc@kemri-wellcome.org. Successful applicants will be provided with full transcripts (in the Swahili language). In the event that one requires translation services for the transcripts, this will be agreed upon with the applicants for it to be done within the programme.

### Extended data

figshare: Additional file 1: Interview guide for in-depth interviews among adults living with HIV, healthcare providers and primary caregivers. https://doi.org/10.6084/m9.figshare.20489208.v1 ^43^

Data are available under the terms of the Creative Commons Attribution 4.0 International license (CC-BY 4.0).
