# Supplementary material for: Strategies for improving mental health and wellbeing used by adults ageing with HIV from the Kenyan coast: a qualitative exploration
Source: Wellcome Open Res. 2023 Jun 22;7:221. Originally published 2022 Aug 26. [Version 2] doi: 10.12688/wellcomeopenres.18212.2 (PMC10320323; doi:10.12688/wellcomeopenres.18212.2)
Supplement: Peer review history

# Peer review: Reviewer response for version 2

**DOI:** 10.21956/wellcomeopenres.21716.r60990

**Published:** 2023-07-04

**Authors:** Jepchirchir Kiplagat

**Affiliations:** 1 Academic Model Providing Access to Healthcare (AMPATH), College of Health Sciences, Moi University, Eldoret, Kenya

The authors have responded to the comments to my satisfaction and would recommend indexing of the manuscript.

Is the work clearly and accurately presented and does it cite the current literature?

Yes

If applicable, is the statistical analysis and its interpretation appropriate?

Not applicable

Are all the source data underlying the results available to ensure full reproducibility?

Partly

Is the study design appropriate and is the work technically sound?

Yes

Are the conclusions drawn adequately supported by the results?

Yes

Are sufficient details of methods and analysis provided to allow replication by others?

Partly

Reviewer Expertise:

HIV and ageing

I confirm that I have read this submission and believe that I have an appropriate level of expertise to confirm that it is of an acceptable scientific standard.

# Peer review: Reviewer response for version 1

**DOI:** 10.21956/wellcomeopenres.20191.r56999

**Published:** 2023-05-22

**Authors:** Marie-Josée Brouillette

**Affiliations:** 1 McGill University, Montreal, Québec, Canada

This qualitative study explores the coping strategies used by Kenyan older adults living with HIV (OALWH) to improve their mental health and wellbeing. Semi-structured in-depth interviews were conducted with 56 participants that included OALWH, healthcare providers and primary caregivers in Kilifi County, a rural area. The needs of OALWH are of increasing interest and this paper is very pertinent, It is well written and clear.

 A few comments for consideration by the authors:

1. In the intro, the authors state: ‘ *The strengths approach encourages policymakers and health professionals to look beyond disease states and approach ageing with HIV as a process to maximize a person’s well-being by strengthening positive factors, e.g. adaptive coping skills.*’; could the authors expand on the implication of their findings for policy makers and specific implications for HC professionals, beyond general statements? For example, the fact the patients did not take their medication when they went without food, was it on response to instruction from the HC professionals and do these instructions have to be changed?
2. It is unclear why the sample included healthcare providers: can you please justify?
3. Recruitment. The sample was recruited using a purposive sampling technique to maximize diversity in respondents’ characteristics, e.g. age and sex., and participants were recruited by trained study staff in liaison with a community health volunteer specialized HIV clinic. All those approached agreed to participate. About 40% of Kilifi residents do not have formal education yet the sample for this study, most (82%) had up to a primary level of education. Is it possible that, while participants were of diverse age and sex, they overwhelmingly represent the more educated, more vocal and open members of the HIV community? The leaders? The narratives are in general quite positive, sometimes surprisingly so. This is the kind of language often heard from leaders in AIDS service organizations, and the section on generativity suggests this as many were in fact leaders. Could the authors comment on how representative of their sample might be?
4. The paper mentions that community-based organizations dedicated to the health and well-being of OALWH did not come up in their discussions. Although the services provided seem pertinent, they were not mentioned. This is an area that may have implications for those groups: what is to be understood from this lack of mention? Is this related to concerns about confidentiality? Lack of pertinence?

Is the work clearly and accurately presented and does it cite the current literature?

Yes

If applicable, is the statistical analysis and its interpretation appropriate?

Yes

Are all the source data underlying the results available to ensure full reproducibility?

Yes

Is the study design appropriate and is the work technically sound?

Yes

Are the conclusions drawn adequately supported by the results?

Yes

Are sufficient details of methods and analysis provided to allow replication by others?

Yes

Reviewer Expertise:

HIV, cognition and quality of life of older adults with HIV

I confirm that I have read this submission and believe that I have an appropriate level of expertise to confirm that it is of an acceptable scientific standard.

# Peer review: Reviewer response for version 1

**DOI:** 10.21956/wellcomeopenres.20191.r52185

**Published:** 2022-10-06

**Authors:** Jepchirchir Kiplagat

**Affiliations:** 1 Academic Model Providing Access to Healthcare (AMPATH), College of Health Sciences, Moi University, Eldoret, Kenya

This is a very interesting paper addressing some of the concern care providers and policy makers should be aware on aging population. This qualitative exploratory study provides relevant information for understanding coping mechanisms used by aging population with HIV to improve their mental health and wellbeing. While this is timely and uncovers ways of improving care for the aging HIV population, the manuscript requires improvement in some areas before its final publication. 

**Title:**

 The title is clear. May want to include the setting of the study as this is key. 

**Abstract:**

 The abstract is detailed and provides a clear and concise.  

**Introduction:**

 The introduction covers relevant information to the study. Sentence 2 in the first paragraph is too long and needs rephrasing. Reference 6 within the first paragraph does not speak to SSA’s limited research. There are two sentences following each other starting with the word ‘Despite’ and need rephrasing too. The last paragraph in the introduction section should be moved to methods.

**Methods:**

 The method section is relatively well done. However, it needs to be clear what type of qualitative interviews were done for each group – in depth interviews? Key informant interviews? Focus group discussions? The methods including the reporting should follow COREQ guidelines. May need to complete the COREQ form to guide the review.

 The authors mention that over three-quarters of the interviews were conducted in Swahili and others in the local language or English. During the analysis recorded interviews were transcribed verbatim. We are not informed at what point the information was translated. At transcription or after?

**Results:**

 The results including the quotes are well presented. It might be helpful to a reader when a table summarizing participants’ characteristics is included. Remove the quantifying word under section on self-care first paragraph where it starts with...a good number of the... you may want to reword to read ... some self-care strategies were .....

 Under generativity, the second sentence begins to discuss the results. Would leave that sentence out and provide it under discussion. There is also a sentence under ‘Social connectedness/support systems’, second paragraph, the second to last sentence that read.... “However, such services tended to vary from one HIV clinic to another.....” This sentence denotes that data was collected from multiple clinics, when in the methods, the authors are clear that the data was collected from Kilifi County Hospital HIV clinic.

**Discussion:**

 This section looks ok as is written. Demonstrate a clear engagement with literature in the field. However, there is a moderate amount of repetitions in the discussion that is not necessary/does not provide any meaning...For example sentence under self-care “...Although existing evidence provides only modest evidence of effectiveness of these strategies, most of these strategies were reported to be effective in promoting mental health and wellbeing among OALWH” First, this sentence needs to be qualified by referencing, and two, ‘these’ strategies need to be clarified.

 Strengths and limitation allude to minimizing potential bias by maintaining reflexivity. How was this done? What potential beliefs/biases did the researcher have in data collection?

**Conclusion:**

 The conclusion is focused to the study findings. However, some statements sound accusative, for example –“Further engagement with traditional healers/prophets may be warranted to mitigate the present obstacles and dangers some of these individuals present to the OALWH”. This statement could be toned down while maintaining what needs to be communicated.

**Data** **availability:**

 Indicate that the transcripts are in Kiswahili. It is unclear how this study was able to complete the analysis and present the data in English if the information was not translated.

Is the work clearly and accurately presented and does it cite the current literature?

Yes

If applicable, is the statistical analysis and its interpretation appropriate?

Not applicable

Are all the source data underlying the results available to ensure full reproducibility?

Partly

Is the study design appropriate and is the work technically sound?

Yes

Are the conclusions drawn adequately supported by the results?

Yes

Are sufficient details of methods and analysis provided to allow replication by others?

Partly

Reviewer Expertise:

HIV and ageing

I confirm that I have read this submission and believe that I have an appropriate level of expertise to confirm that it is of an acceptable scientific standard, however I have significant reservations, as outlined above.
